# The impact of pre-stroke formal education on language test performance in aphasic and non-aphasic stroke survivors

**DOI:** 10.1080/02687038.2024.2434864

**Published:** 2024-12-05

**Authors:** Sophie M. Roberts, Rachel Bruce, Thomas M. H. Hope, Sharon Geva, Storm Anderson, Hayley Woodgate, Kate Ledingham, Andrea Gajardo-Vidal, Diego L. Lorca-Puls, Jennifer T. Crinion, Alexander P. Leff, David W. Green, Cathy J. Price

**Affiliations:** a Department of Imaging Neuroscience, University College London, London, UK; b School of Psychology and Sports Sciences, Anglia Ruskin University, Cambridge, UK; c Centro de Investigación en Complejidad Social (CICS), Facultad de Gobierno, Universidad del Desarrollo, Concepción, Chile; d Sección de Neurología, Departamento de Especialidades, Facultad de Medicina, Universidad de Concepción, Concepción, Chile; e Institute of Cognitive Neuroscience, University College London, London, UK; f Department of Brain Repair and Rehabilitation, University College London, London, UK; g Department of Experimental Psychology, University College London, London, UK

**Keywords:** Aphasia, education, assessment, recovery

## Abstract

**Background:**

A greater amount of education is known to positively impact language skills in neurotypical populations, but its influence on language outcomes and recovery after stroke remains unclear.

**Aims:**

This study of 749 stroke survivors, with and without aphasia, investigated (A) which aphasia assessment tasks benefitted most from more pre-stroke education; and whether the effect of education (B) differs for aphasic and non-aphasic participants or other stroke and non-stroke-related variables, and/or (C) facilitates recovery from post-stroke aphasia.

**Methods:**

Participants ranged from one month to 42 years post-stroke. They were assessed using (i) the Comprehensive Aphasia Test (CAT), and (ii) self-report questionnaires that measured speech production, comprehension, reading, and writing at one week and one year post-stroke. Multiple regression analyses investigated the effect of education amount, and its interaction with other variables, on language outcomes and recovery. Bayesian statistics assessed the strength of the evidence for any observed effects. Many variables including lesion size, age at stroke, and initial severity were controlled for.

**Results:**

(A) More years of formal education were associated with better overall language skills, with significant, albeit small effects found for semantic and letter fluency (β = 0.123 and 0.166) and spoken picture description, specifically, the number of words produced (β = 0.085) and grammatical well-formedness (β = 0.087). (B) The benefit of more pre-stroke education was mostly additive with the effects of other variables including initial aphasia severity and left hemisphere lesion size, but was reduced in older participants who had large lesions with severe initial symptoms. Finally, (C) no significant effect of education on language recovery was observed.

**Conclusion:**

More pre-stroke formal education is associated with higher post-stroke language scores on a wide range of tasks for both aphasic and non-aphasic participants, but, in participants with large lesions that cause severe aphasia, this advantage diminishes with age. These results suggest a generic benefit of education on language test performance rather than a specific role of pre-stroke education in aiding language outcomes and recovery. An individual’s educational background should therefore be considered when interpreting assessment scores.

## Introduction

This study investigates whether, how and to what degree the amount of pre-stroke education a person received influences post-stroke speech and language ability and recovery. It focuses on three specific questions in a large cohort of stroke survivors: (A) Which aphasia assessment tasks are most influenced by the amount of pre-stroke education? (B) How does the impact of education vary with other stroke-related and non-stroke factors? and (C) To what extent does pre-stroke education influence post-stroke language recovery? By including stroke survivors with and without aphasia, our study is the first to disentangle how much the effects of pre-stroke education, observed for questions A-C, are explained by (i) the generic benefits of education on language test performance (expected in both aphasic and non-aphasic participants) versus (ii) a specific role of pre-stroke education in aiding language outcomes and recovery (i.e., a greater impact of pre-stroke education on aphasic than non-aphasic participants). Answering these questions is important for understanding whether education should be considered a prognostic factor in aphasia research and clinical practice.

Further and Higher Education, in the UK, refer to formal learning or training that is delivered by trained instructors, and typically takes place at educational institutions. Formal education increases exposure to specialised vocabularies, independent learning, and critical and flexible thinking. It also enhances cognitive, communication and problem-solving skills. The long-term influence of a rich language environment, offered through education, can manifest through better performance on tasks that test verbal fluency, object naming, digit span, and sentence comprehension and production. Numerous studies have demonstrated this in neurotypical populations (see Table S1) including an early study that used language tasks from a popular aphasia assessment (Borod et al., 1980). The advantage of education in neurotypical populations has been observed over multiple studies, despite wide variation in methodological factors such as: the definition of education, the participants’ ethnicities and languages, education systems, and other covariates considered in the analyses.

Although an abundance of evidence has shown that education benefits language abilities in neurotypical populations, very few studies have demonstrated educational benefits on post-stroke language ability. One study, with 173 stroke patients, reported that more education was associated with better reading scores (written word comprehension and oral reading) but not with better auditory comprehension or naming scores (González-Fernández et al., 2011). This was observed after controlling for age, sex, lesion volume and socio-economic status. Another study of 39 participants reported that the initial severity of aphasia was worse when education and occupational status were lower (Connor et al., 2001), and a study of 168 participants associated more education with better auditory comprehension scores in those with chronic aphasia (Lwi et al., 2021). However, other studies have found no significant impact of education on aphasia outcome. For example, a recent and well powered study by Johnson et al. (2022) reported that years of education was not significantly predictive of chronic aphasia severity in 147 participants when other demographic, lesion, and cognitive variables were factored out.

Inconsistency in the effect of education has also been demonstrated in studies that focused on the degree of recovery from post stroke aphasia. Only one study, using a cohort of 235 patients, demonstrated that education influenced post-stroke language recovery (Kim et al., 2019) even after factoring out the influence of age, initial severity and lesion size. This finding motivates further research, particularly as the authors did not explore the education effect in depth, for example to determine which language tasks were most sensitive to education. Nor did they determine whether the effect of education varies with the effects of other variables. In contrast no significant effect of education was observed on aphasia recovery in three much smaller studies: Connor et al. (2001) with 39 patients, Lazar et al. (2008) with 22 patients, and Lwi et al. (2021), with 44 patients.

A benefit of education on language abilities after brain injury may be explained by “cognitive reserve”. Cognitive reserve describes a property of the brain that reduces an individual’s susceptibility to, or compensates for, pathology or injury, by utilising behavioural strategies, or engaging alternative neural networks (Stern et al., 2023). Education is likely to be one of many factors that contribute to cognitive reserve that is acquired throughout the lifetime (along with social engagement, mentally stimulating pursuits, physical activity and genetics). In the dementia literature, it has been argued that greater cognitive reserve may delay the onset of cognitive decline (Meng et al., 2012; Stern, 2009, 2012; Stern et al., 2020) or the detection of cognitive decline when premorbid abilities were very high. If greater cognitive reserve renders individuals more resilient to cognitive decline in the face of neurodegeneration, it might also be the case that cognitive reserve protects against cognitive impairment following stroke. For example, post-stroke aphasia outcome and/or recovery may be better in people who received more education and who, as a result, can capitalise on more efficient cognitive strategies. These individuals would likely also benefit from higher socioeconomic status (Sheridan & McLaughlin, 2016), that may enable greater access to healthcare and treatment, which promote a faster recovery. However, several systematic reviews have shown very inconsistent evidence for the relationship between socioeconomic status and recovery, and highlighted the need for more studies (Contador et al., 2023; Laura & Roza, 2022; Nunnari et al., 2014; Rosenich et al., 2020; Tao et al., 2023). Plausibly, the benefit of more education, and cognitive reserve, on language abilities is lost when patients have extensive damage to the neural systems that normally support language recovery. In other words, the benefit of education may depend on one or more of the many lesion- and non-lesion factors that are known to influence aphasia outcome and recovery.

Alternatively, a benefit of education on post-stroke language outcomes could reflect the generic benefit of education on language abilities that is observed in neurotypical populations. In other words, an advantageous effect of education is present pre-stroke, and detected during language assessment; but pre-stroke language abilities are not measured in aphasic stroke survivors, meaning effects of education that are observed on post-stroke language abilities could be misinterpreted. Testing this hypothesis would only be possible by including a control group (without stroke or aphasia).

Returning now to the questions addressed by the current study, we make the following hypotheses. First, regarding Question A, we hypothesised that the benefit of education would be stronger on Reading and Written comprehension tests than Naming or Repetition as found by González-fernández et al. (2011), and also stronger on Picture description than Naming. Our reasoning is that Naming and Repetition are relatively simple tasks, which use common vocabulary, and in the case of Repetition, do not necessarily require semantic knowledge of the items. Even knowledge of the items characterised as “low frequency” (e.g., *pineapple, drum*) may not depend on the amount of prior education undertaken. Conversely, Reading and Written comprehension utilise literacy skills that are more explicitly associated with formal education, as well as with experience of examination and assessment. Picture description allows multiple different words to be used for the same target (e.g., *man, father, grandfather)* whereas correct Naming responses are typically limited to one specific word. Picture description also benefits from syntactic variety and better grammar (Zanichelli et al., 2020) which are enhanced by more education. Second, regarding Question B, we hypothesised that more education would have a reduced effect in participants who had worse post-stroke language ability because, in these participants, other factors (e.g., lesion size) are stronger determinants of language ability post-stroke, potentially overriding the pre-existing benefit of education. Despite this variability, our third hypothesis regarding Question C was that more education would enhance the degree of post-stroke language recovery, measured both during the first year and well into the chronic stage of recovery. This hypothesis was motivated by the findings of Kim et al. (2019), even though this study focused on recovery only within the first year post-stroke.

## Methods

The study was approved by the London Queen Square Research Ethics Committee (study reference number 13/LO/1515). All participants gave written informed consent (or assent via a consultee) to participate.

### Participant selection

Participants were selected from the Predicting Language Outcome and Recovery After Stroke (PLORAS) database (Seghier et al., 2016). The database holds demographic, behavioural and lesion data for over 1500 stroke patients, who are recruited from various locations around the UK, including via the National Institute for Health and Care Research (NIHR) Clinical Research Network, stroke groups and conferences, and word-of-mouth recommendations. Participants were selected if they (1) were native English speakers, (2) had a clinically-diagnosed stroke in either the left or right hemisphere, and (3) had no other neurological disorders that might influence their speech and language abilities. They were excluded if they (a) had unknown initial speaking ability (*n* = 212), (b) were too medically unwell (e.g., in a coma) to attempt speech production one week post-stroke (*n* = 145), (c) had inconsistent self-reported language ability compared to their objectively assessed language abilities (*n* = 12), or (d) had an unknown (*n* = 6) or outlying amount of pre-stroke education (*n* = 5; with 14–16 years). These criteria identified 749 eligible participants (see Table 1).

**Table 1.** Participant characteristics.

		Less education	More education
		(*n* = 413)	(*n* = 336)
Years of education since age 14	Mean (SD)	2 (1)	8 (2)
	Range	0-4	5-13
Initial speaking severity	Severe (%)	145 (35)	98 (29)
	Moderate (%)	59 (14)	59 (18)
	Mild (%)	131 (32)	111 (33)
	Normal (%)	78 (19)	68 (20)
Sex	Male (%)	287 (69)	231 (69)
	Female (%)	126 (31)	105 (31)
Pre-stroke handedness	Right (%)	356 (86)	292 (87)
	Left (%)	48 (12)	29 (9)
	Ambidextrous (%)	9 (2)	15 (4)
Age at stroke	Mean (SD)	58 (12.5)	57 (14)
	Range	17-88	18-98
Years post-stroke of CAT	Mean (SD)	4.2 (5.2)	3.8 (4.5)
	Range	0.1-42.4	0.1-36.0
Lesioned hemisphere	Left (%)	211 (51)	172 (51)
	Right (%)	111 (27)	90 (27)
	Both (%)	73 (18)	61 (18)
	Undetected^a^ (%)	18 (4)	13 (4)
Left hemisphere lesion size (cm^3^)	Mean (SD)	33 (62)	28 (53)
	Range	0-429	0-355
Right hemisphere lesion size (cm^3^)	Mean (SD)	19 (43)	15 (43)
	Range	0-280	0-357
Developmental dyslexia status	Formal diagnosis (%)	6 (1)	8 (2)
	None (%)	232 (56)	193 (57)
	Unknown (%)	175 (42)	135 (40)
Semantic memory score^b^	Impaired (%)	25 (6)	12 (4)
	Within normal limits (%)	388 (94)	323 (96)

Legend.

a “Undetected” lesions were those that were not detectible on the research scans available.

b Score threshold: T score of ≤ 47 indicates impairment, and of ≥ 51 indicates within normal limits. One participant did not receive a score for this subtest.

A complete set of behavioural, demographic and lesion data was available for most of the participants. However, 182 participants had missing data for several variables (initial reading/comprehension severity, hearing, vision, and CAT task scores), and were excluded from the relevant analyses; see the Statistical Analyses section for more details. The impact of excluding these participants is considered in the Discussion.

### Pre-stroke education

Education amount was measured, via participant reports, as the number of years of formal education completed since the age of 14. This represented the “least” amount of education completed by a participant in the sample. This study’s definition of formal education includes subject-based qualifications such as, within the UK education system, GCSEs, AS and A levels (or equivalent qualifications), and undergraduate and post-graduate degrees and diplomas. Apprenticeships, work-related qualifications and continuing professional development courses are not included, as they typically involve different learning content, style and assessment methods compared to school or university-based education, which relies more heavily on written material and examination. The potential implications of their exclusion from our definition of “formal education” is considered in the Discussion. The number of years of education was primarily treated as a continuous variable, but was also used to allocate participants into groups for some analyses and illustrations. “Less” education included participants who underwent education up to age 18 (i.e., A Levels or equivalent), and “More” education included participants who continued formal education beyond age 18 (i.e., university).

### Language outcomes

All participants were assessed with a standardised objective language and cognitive assessment – the Comprehensive Aphasia Test, or CAT (Swinburn et al., 2004). The timing of the CAT assessment was different for each participant – ranging from 17 days to 42 years post-stroke – depending on when the participant was recruited to the study, and/or when they felt sufficiently recovered to take part. The vast majority of participants were tested in the chronic period (>6 months post-stroke), however a minority (*n* = 80) took part in the first 6 months post-stroke. To compare performance on each of the tasks (with varying demands and difficulty), raw scores are converted through a non-linear transformation into T-scores, which represent how well the participant performed relative to an independent sample of patients with aphasia. Lower scores indicate poorer performance. The focus of the study was on 19 tasks, whose T scores are integrated into 6 “summary” T scores for different language modalities, described below. In addition, these six summary scores were averaged to construct an “overall language ability” score (as in Winans-Mitrik et al., 2014).

1. Naming combined scores from: object naming, action naming, semantic fluency and letter fluency. Object naming involved naming 24 objects (e.g., *frog*) from simple line drawings; action naming involved naming 5 actions (e.g., *typing*); semantic fluency involved producing as many animal names as possible within one minute; and letter fluency involved producing as many s-initial words as possible within one minute. In the CAT, the raw scores for semantic and letter fluency are combined into one T score for overall “word fluency”, however in this study, their raw scores were analysed individually, in order to determine any differences in how education influenced each fluency component.
2. Repetition combined scores from: repeating 16 words (e.g., *plant*), 5 complex words (e.g., *defrosted*), 5 nonwords (e.g., *gart*), digit strings (increasing from 2 to 7 numbers), and sentences (increasing from 3 to 6 content words).
3. Reading combined scores from: reading aloud 24 simple words (e.g., *family*), 3 complex words (e.g., *informative*), 3 function words (e.g., *but*) and 5 nonwords (e.g., *fask*).
4. Spoken picture description involved describing the objects, characters and actions in a scene. The score comprises the total number of appropriate and inappropriate information-carrying words produced in one minute, the grammatical well-formedness and syntactic variety of the sentences, and the speed of production (assessed according to whether the participant spoke with any lengthy delays or hesitations).
5. Spoken Comprehension combined scores from spoken word-to-picture matching, spoken sentence-to-picture matching, and spoken paragraph understanding, which involve an auditory stimulus (e.g., *mouse*) and require a correct point-response in the presence of phonological, semantic and unrelated distractors (*house*, *rabbit*, *church*).
6. Written Comprehension combined scores from written word-to-picture matching and written sentence-to-picture matching, both of which involve a written stimulus (e.g., *rocket*) and require a correct point-response in the presence of phonological, semantic and unrelated distractors (*pocket*, *aeroplane*, *sleeve*).

**Figure 1.**
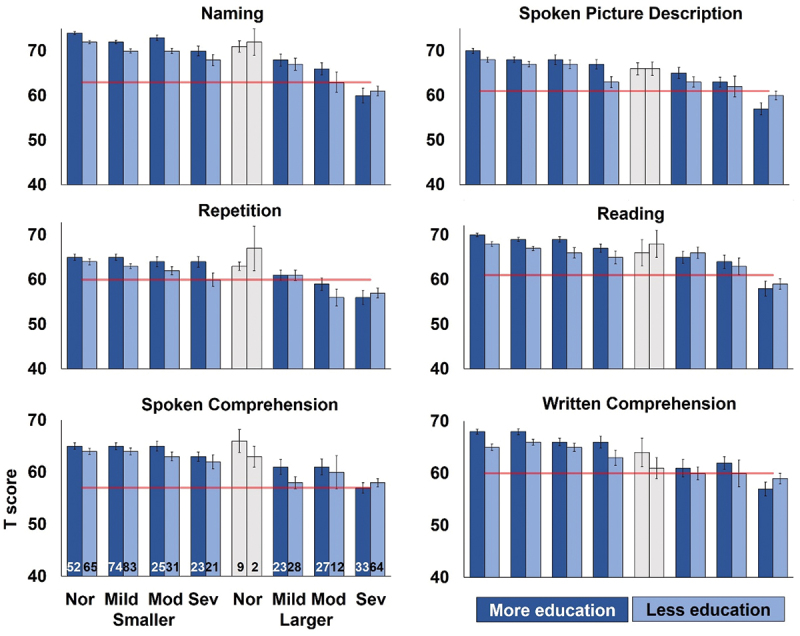
The effect of education on six CAT summary scores.

**Legend**: CAT T scores for the six summary scores, grouped by education amount, initial speaking ability and lesion size. Grey = fewer than 10 participants (i.e., not well-sampled). Nor, Mild, Mod, Sev = Normal, Mild aphasia, Moderate aphasia and Severe aphasia. Smaller = smaller left hemisphere lesions (0–3 cm^3^). Larger = larger left hemisphere lesions (more than 9 cm^3^). Group numbers are shown in the plot for spoken comprehension (bottom left). See Methods for details of group matching. Aphasia score thresholds for each task are indicated by red horizontal lines.

An aphasia/normal threshold for each language summary skill, specified in the CAT manual, indicates whether a T score falls into the aphasic or the normal range (e.g., ≤62 and ≥ 63 for Naming; illustrated in Figure 1). As the overall language ability score is not part of the CAT, no control range was available to calculate the aphasia threshold score.

### Initial aphasia severity

Each participant was assigned a one week post-stroke initial aphasia severity rating derived from an in-house retrospective participant-reported outcome measure of language ability. The 4 severity categories were: “Severe” (unable to produce any speech, or only able to produce automatic speech), “Moderate” (able to produce single words and phrases), “Mild” (able to produce lexically meaningful short sentences), and “Normal” (no stroke-related difficulties with language production). Research speech and language therapists supported participants to provide an accurate report of their difficulties (for example, differentiating fluent from non-fluent aphasia). The ratings were repeated for speaking, comprehension, reading, and writing. A participant could therefore be assigned to different initial severity groups for different language functions (e.g., if they had speaking difficulties, but intact comprehension). Participants rated as Severe were conscious and medically capable of attempting to speak – but may not have been able to produce any words, due to aphasia (and/or dysarthria/apraxia).

### Lesion data

High-resolution (1 mm × 1 mm x 1 mm), whole brain T1-weighted structural brain images were acquired for all participants on research-dedicated scanners at the Wellcome Centre for Human Neuroimaging and the Birkbeck-UCL Centre for Neuroimaging. The MRI scanners used were all from Siemens Healthcare (Erlangen, Germany). Brain scans were typically obtained on the same day as CAT assessment (ranging from 2 weeks to 42 years post-stroke).

Using standard procedures within SPM software (Wellcome Centre for Human Neuroimaging, London, UK; https://www.fil.ion.ucl.ac.uk/spm/), running in MATLAB environment (2018a Mathworks, Sherbon, MA, USA), each T1-weighted image was spatially normalised (to the MNI template) and converted into a quantitative assessment of structural abnormality that is independent of the scanner used (Seghier et al., 2008). At each voxel, this “fuzzy” lesion image encodes the degree of abnormality (U) on a continuous scale from 0 (completely normal) to 1 (completely abnormal) relative to normative data from 64 neurologically-intact controls that were collected on the same scanners used for image acquisition in the current study. To delineate the lesions and estimate lesion volume, each fuzzy lesion image was thresholded into a “binary” lesion image (i.e., presence or absence of a lesion, at each voxel). As recommended in Seghier et al. (2008), the abnormality/lesion threshold for binary images was set using a U value of 0.3. The resulting binary lesion images were used to calculate left and right hemisphere lesion size separately.

### Demographic and assessment variables

Study questionnaires capture each participant’s biological sex, pre-stroke handedness, developmental dyslexia status, date of birth, and date of stroke (see Table 1). Age at stroke was subsequently calculated from date of birth and date of stroke. Semantic impairments were measured using the CAT “semantic memory” task which is, more precisely, a nonverbal “semantic picture matching” task. It involves matching a target picture (e.g., *monkey)* to one of four surrounding choice pictures (e.g., *banana, pear, chocolate* and *envelope*) and measures the ability to perceive pictures, recognise objects, and identify semantic links. Impairments on this task may impact any language tasks which involve visual or conceptual stimuli (including both pictures, and real words presented visually or aurally), so these task scores were used to control for such impairments. To control for perceptual abilities that might impact the ability to perceive the assessment stimuli, participants’ hearing and vision were corrected wherever possible (e.g., with hearing aids, glasses), and their own hearing and vision status was self-reported at the point of assessment.

## Statistical analyses

Three analyses were designed to answer each question of interest (A, B and C).

### A. Which language scores are most sensitive to amount of pre-stroke education?

This question is approached in three steps: (1) Is there a main effect of education on overall language ability score? (2) Which summary scores drive this effect? (3) Which individual tasks drive effects of education in the summary scores? As there is only one statistical analysis in the first step, there is no need to correct for multiple comparisons. The second and third steps involve 6 and 24 different analyses (one for each summary and individual task score). If the main effect in step one is significant, the second and third steps can be considered as descriptive, reducing the need for multiple comparison corrections. Nevertheless, in the Table legends, we note which summary and task scores would survive correction for multiple comparisons if they were considered independently.

Each of the analyses described was conducted using multiple linear regression, performed in IBM SPSS Statistics (version 28.0). The following covariates were included in all analyses: age at stroke, time post-stroke of the CAT assessment, left and right hemisphere lesion size, sex, pre-stroke handedness and developmental dyslexia status. The inclusion of other covariates depended on the language task being considered. For example, initial speaking ability was included in the analyses of speaking scores; initial comprehension ability in the analysis of auditory comprehension scores, initial reading ability in the analysis of reading scores, hearing status in the analysis of tasks that used auditory stimuli, visual status in the analysis of tasks that used visual stimuli, and semantic picture matching score in the tasks that required object recognition or semantic knowledge (i.e., any task that used real words/objects, rather than non-words). As some participants had missing data, different participant numbers were included in different analyses; see Table S2 for details of all covariates and Table 2 for the participant number in each analysis. We are confident that there was minimal multicollinearity in the data, because no analysis yielded Tolerance values of less than 0.1, nor Variance Inflation Factors (VIF) of greater than 10.

**Table 2.** The effect of education on post-stroke CAT scores.

		Multiple linear regression			Bayesian regression	
Outcome measure	N	R^2^	β	p	Log BF	Evidence strength
**Overall language ability**	569	0.632	0.075	0.004	0.29	Anecdotal
**Naming:**	741	0.580	0.079	0.001*	0.74	Strong
Semantic fluency	748	0.423	0.123	<0.001*	2.48	Decisive
Letter fluency	748	0.384	0.166	<0.001*	5.27	Decisive
Object naming	741	0.473	0.014	ns		
Action naming	741	0.455	0.024	ns		
**Repetition:**	743	0.478	0.054	0.047		
Word repetition	743	0.380	−0.017	ns		
Complex word repetition	743	0.387	−0.021	ns		
Nonword repetition	743	0.342	0.072	0.019		
Digit span	744	0.391	0.076	0.010		
Sentence repetition	743	0.432	0.012	ns		
**Reading:**	572	0.444	0.058	ns		
Reading words	573	0.435	0.043	ns		
Reading complex words	572	0.431	0.027	ns		
Reading function words	572	0.188	0.044	ns		
Reading nonwords	572	0.394	0.076	0.022		
**Spoken picture description:**	741	0.516	0.059	0.024		
Total number of words	741	0.415	0.085	0.003	0.36	Anecdotal
Number of appropriate information-carrying words	741	0.441	0.073	0.009		
Number of inappropriate information-carrying words	741	0.134	0.087	0.013		
Grammatical well-formedness	741	0.433	0.087	0.002	0.48	Anecdotal
Syntactic variety	741	0.418	0.052	ns		
Speed	741	0.438	0.025	ns		
**Spoken comprehension:**	709	0.388	0.066	0.029		
Spoken word-to-picture	711	0.272	0.039	ns		
Spoken sentence-to-picture	710	0.400	0.073	0.014		
Spoken paragraph	710	0.118	0.016	ns		
**Written comprehension:**	573	0.525	0.076	0.010		
Written word-to-picture	574	0.339	0.054	ns		
Written sentence-to-picture	573	0.526	0.067	0.022		

Legend: Effects of education on overall language ability, and summary and individual task scores from the CAT, using (i) multiple linear regression and (ii) Bayesian regression. The number in each analysis varies due to missing data for certain covariates. R2 = for the whole model. ns = not significant at a threshold of *p* = <0.05. BF = Bayes Factor: Positive BF supports an effect of education; Evidence strength: >2=decisive; 1.5–2 = very strong; 1–1.5 = strong; 0.5–1 = substantial; 0–0.5 = anecdotal (Jeffreys, 1961). Negative BF refutes the effect (empty cells indicate negative BF). * = significant after correction for multiple comparisons.

Bayesian statistics were used to validate the results further by estimating evidence for or against an effect of education on each language measure. This was done by conducting a “naïve Bayesian linear regression” (Lee & Wagenmakers, 2014) in SPSS; which uses default hyperparameters, and thus uninformative priors. This method involved first repeating the multiple linear regression for each outcome, omitting education, to obtain the standardised residuals, and then regressing these residuals with education. The Bayes Factors were then converted into Log BF. A negative log BF indicates evidence for no effect of education, whereas a positive log BF indicates evidence for an effect of education. The strength of evidence ranges from “anecdotal” to “decisive” (Jeffreys, 1961), see Table 2 legend for categories of evidence strength.

### B. How does the effect of education depend on other variables?

Moderation analyses were used to investigate potential interactions between education and six other variables of interest. The interaction terms were created by multiplying “years of education” with each variable: age at stroke, time post-stroke, initial aphasia severity, semantic picture matching scores, and right and left hemisphere lesion size. Two-way interaction terms were added into a second block of the regression; three-way interaction terms were added into a third block of the regression (along with the relevant two-way interaction terms) and four-way interaction terms were added into a fourth block of the regression (along with the relevant two-way and three-way interaction terms). The first block of the model contained the same covariates as the main analyses. These moderation analyses were conducted first for overall language ability, then repeated as post-hoc tests for each of the six summary scores.

Post hoc analyses of significant interactions were conducted by testing whether there was an effect of education in different subgroups of participants. For age at stroke, the subgroups were created using the median age: the “younger” group were 17 to 57.88 years (*n* = 375) and the “older” group were 57.93 to 99 years (*n* = 374). For initial aphasia severity, the three categories were used (Normal, *n* = 146; Mild, *n* = 242; Moderate & Severe, *n* = 361). Participants with Moderate initial severity were included in the Severe aphasia groups to increase group sizes. For left hemisphere lesion size, two grouping approaches were considered. First, we identified the median left hemisphere lesion size of the full sample (3.3 cm^3^); and created two subgroups either side of this median (“larger”, *n* = 375, and “smaller”, *n* = 374). Each subgroup was then further split by pre-stroke education amount (“less” versus “more”). However, the educational groups within the larger and smaller lesion categories were not matched for either lesion size or time-post stroke, raising the possibility that higher scores for more versus less education could reflect longer time post-stroke (i.e., more time to recover) or smaller lesions (i.e., less damage and better recovery capacity) instead of a genuine benefit of education. To mitigate this, we removed 177 participants from the “larger” subgroups – leaving 198 participants, whose high versus low education groups were more closely matched for lesion size and time post-stroke.

The second lesion size approach illustrated the interactive effects between education, lesion size, age and initial aphasia severity. Subgroups were created for participants with left hemisphere lesions of less than 1 cm^3^, and greater than 1 cm^3^ (Figure 3).

### C. Does amount of education influence the degree of recovery?

Two methods were used to investigate the relationship between education and recovery.

**CAT scores**: An effect of education on recovery would be indicated by a significant interaction between education and time post-stroke (investigated in section B) with post hoc tests showing that, as time post-stroke increased, the advantage of more education also increased. However, as the CAT scores were mostly acquired years post-stroke (see Table 1), this analysis will not be sensitive to the influence of education on early recovery (i.e., in the first year post stroke). To determine whether an effect of education on recovery depended on other variables, we examined the three- and four-way interactions between education, time post-stroke and (i) left hemisphere lesion size, (ii) age and (iii) initial severity.

**Self-rated scores**: A “recovery score” was calculated for each participant by dividing [actual improvement at one year post-stroke] by [improvement potential]. Actual improvement is the number of ability levels by which a participant increases between one week and one year (Hope et al., 2019; Kim et al., 2019). For example, improving from Severe to Mild equates to an actual improvement of 2 (Severe to Moderate and Moderate to Mild). Improvement potential is the total number of severity levels available to increase by. This is maximum for participants with Severe initial aphasia symptoms who can move up three levels (Severe to Moderate, Moderate to Mild and Mild to Normal). Conversely, those with mild initial aphasia symptoms can only move up one level (Mild to Normal). In line with others (Lazar et al., 2010; Marchi et al., 2017) and with awareness of the pitfalls (Bonkhoff et al., 2020; Bowman et al., 2021; Hope et al., 2019), the recovery score was calculated as a proportion; for example, a participant who improved by 2 out of 3 possible performance increases would receive a recovery score of 0.66. This method of measuring recovery score enables all participants to be analysed together, regardless of their initial severity. Recovery score was calculated for each of the four language skills that we had assessed (speaking, comprehension, reading, and writing). The mean of these recovery scores was also calculated for each participant. Multiple linear regression was used to investigate the relationship between education and recovery score, when left and right hemisphere lesion size, age, sex, and handedness were factored out. As with the CAT recovery analyses, two- and three-way interactions between education and (i) age, (ii) left hemisphere and (iii) right hemisphere lesion size were examined to determine whether an effect of education on recovery depended on these other variables.

## Results

### A. Which language scores are most sensitive to pre-stroke education amount?

Across all aphasic and non-aphasic participants, the multiple regression analysis revealed that years of education had a significant, positive effect on overall language ability (β = 0.075, *p* = 0.004), suggesting that, after factoring out the effect of other variables, each additional year of education was associated with a score increase of 0.8 (Table 2). This advantage of more education was also observed, independently, on all six summary scores (Figure 1).

**Figure 2.**
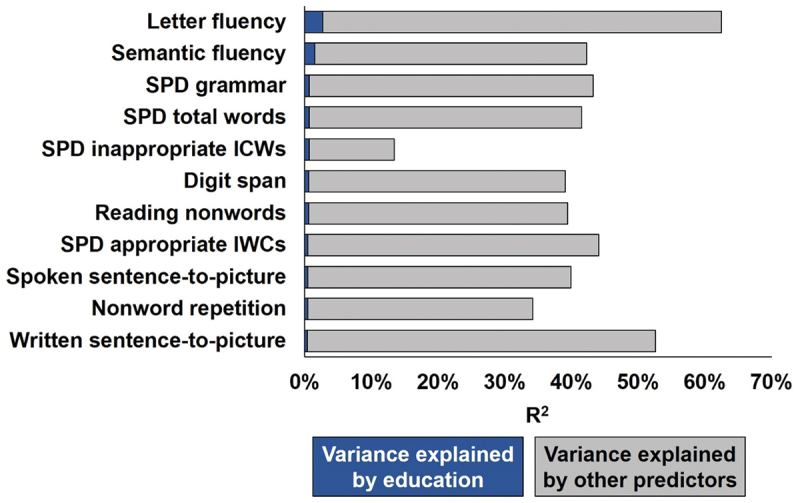
The variance in CAT task scores explained by (i) education amount and (ii) other variables.

As detailed in Table 2 and Figure 2, the effects of education were strongest for: (1) letter fluency and semantic fluency within Naming, (2) digit span and nonword repetition within Repetition, (3) nonword reading within Reading, (4) the total number of words produced during spoken picture description and their grammatical well-formedness, and (5) sentence comprehension within both spoken and written Comprehension scores. The amount of variance explained by education, when education was the only regressor, was 4.1% for letter fluency, 3% for semantic fluency and less than 2% for all other scores (see Table S3 for all other tasks).

### B. How does the effect of education depend on other variables?

**Figure 3.**
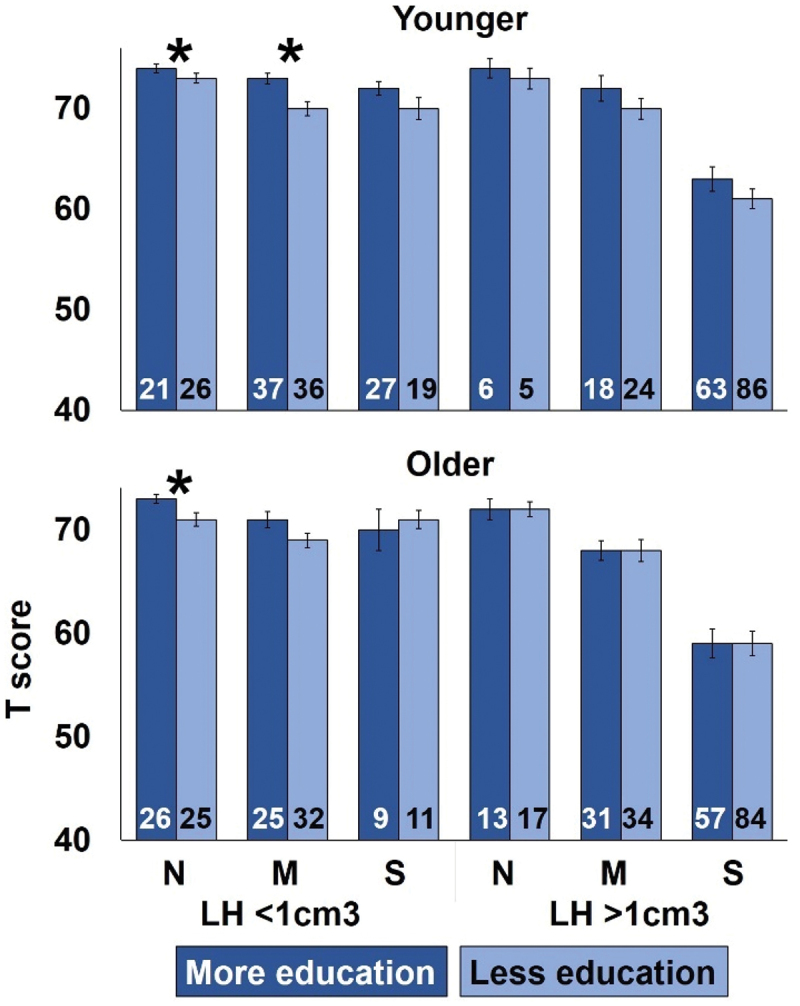
Interaction between education, age, lesion size and initial speaking ability on naming scores in 732 participants.

Legend: Average Naming (summary) T scores for participants who were younger (top) or older (bottom), grouped by education amount, initial speaking ability and left hemisphere damage. N, M, S = Normal, Mild aphasia, and Severe aphasia. Participants with Moderate initial severity are included in the Severe aphasia groups to increase group sizes. LH <1 cm^3^ = small left hemisphere lesions and/or larger right hemisphere lesions. LH >1 cm^3^ = left hemisphere lesions that were larger than 1 cm^3,^ irrespective of right hemisphere damage. * denotes a significant effect of education across each subgroup pair, at a significance level of *p* = <0.05.

The moderation analyses indicated that the benefit of more education was dependent on age for most outcome measures (except for spoken picture description, spoken comprehension and written comprehension), with post hoc tests indicating that the benefit of education was primarily driven by younger participants (Table S4). For naming and spoken picture description, there was a significant four-way interaction between education, age, left hemisphere lesion size and initial speaking ability (Table S4). This four-way interaction arose because a benefit of more education was observed for younger participants irrespective of lesion size or initial speaking ability (upper part of Figure 3); whereas in older participants, a benefit of education was only observed when left hemisphere damage was less than 1 cm^3^ (i.e., very small) and initial speaking ability was normal or mildly impaired (lower part of Figure 3). In contrast, no benefit of education was observed on naming scores for older participants whose lesions were larger than 1 cm^3^, irrespective of initial speaking ability.

There were no other significant interactions between education and other variables on overall language ability or any of the summary scores or individual task scores, after correction for multiple comparisons. Indeed, the benefit of education was remarkably robust across groups of participants with different lesion sizes and different initial severities, with the exception that, for participants with large lesions and severe initial aphasia, all scores were low, irrespective of education amount (Figure 1).

### C. The effect of education on the degree of recovery

**CAT scores**: Evidence of recovery was observed as a significant positive effect of time-post stroke on overall language ability (*p* = 0.002, β = 0.088), indicating that with every additional year, scores increased by almost 1 point. Although the interaction between education and time post-stroke did not reach significance, there was a significant three-way interaction between education, time-post stroke and left hemisphere lesion size (*p* = 0.015). However, this did not reflect education facilitating recovery; it was driven by a decline in language scores, over time, in participants who had large left hemisphere lesions and more education. This observation is likely to reflect sampling bias because of a combination of three study factors: (a) the effect of time post-stroke is across subjects in this analysis; (b) our study requires participants to visit our research centre in London; which is, critically (c) more difficult for those with large left hemisphere lesions and severe impairments. Participants with large lesions are therefore more likely to be recruited early post-stroke if they have mild rather than severe impairments.

**Self-rated scores**: Recovery score was not significantly associated with the amount of education for any of the 4 individual language skills or their mean. No interactions were observed between education and any other variables.

## Discussion

Prior studies have demonstrated that performance on language assessments is sensitive to the amount of education undertaken in neurotypical people (see Introduction and Table S1). The current study is the first to show that the benefit of more education on language performance is remarkably similar for both aphasic and non-aphasic participants. This finding is important, because it emphasises the potential sensitivity of aphasia assessments to amount of education, rather than demonstrating that more education specifically benefits aphasia outcome and recovery. The discussion below addresses our three study questions (a-c), the implications of the findings, study limitations and future directions.

### A. Which CAT tasks are most sensitive to education amount, and why?

The benefit of education was strongest for semantic and letter fluency; but also highly significant for nonword reading, nonword repetition, digit span, written sentence comprehension, spoken sentence comprehension and the number of words produced during spoken picture description, and their grammatical well-formedness (see Results and Table S3). Compared to González-Fernández et al. (2011), our findings (i) broaden the range of language tasks that may benefit from education to include spoken comprehension (also consistent with Lwi et al. (2021)), digit span, nonword repetition and picture description, (ii) are consistent in showing no advantage of education on word repetition or object naming and (iii) only found a benefit of education on nonword reading, not word reading. Further studies are now required to determine if the effect of education on reading words depends on stimulus properties (e.g., word frequency, orthographic to phonological regularity, concreteness etc.), or other factors.

The generic benefits of education on verbal skills are well recognised. Education enriches vocabulary and exposes people to a wider range of linguistic structures for organising, embellishing, and understanding speech. More education may increase proficiency in language use and enhance the underlying cognitive processes that support language production and comprehension. For example, during spoken picture description, visual perception and working memory are required to perceive the stimuli and hold their contents in memory, semantic information needs to be retrieved and integrated, word-finding difficulties need to be circumvented by retrieving other appropriate words, and speech output needs to be self-monitored and self-corrected. All these processes may benefit from more education. In addition, the ability to perform post-stroke language assessments may also benefit from the increased examination experience associated with education.

Semantic and letter fluency may benefit most from education experience because fluency tasks are facilitated by a range of language and cognitive skills that can be enhanced during education, such as a wide-ranging vocabulary, proficient semantic and phonological retrieval strategies and possibly verbal short-term memory and executive control. For example, cognitive control is required to suppress incorrect responses during both fluency and picture description.

Finally, although the effects of education are highly significant, it is important to note that they only accounted for a small amount of the variance (see Table S3 and Figure 1). This is relevant in the context of the prior studies of education effects in aphasia because such effects may only be reliably detected when large samples are available, as in the current study.

### B. How the effect of education on task performance depends on other variables

Age was the only variable to significantly influence the effect of education. This was observed on: overall language ability, naming, repetition and reading summary scores, but not on spoken picture description or comprehension. In each case, the interaction indicated a sub-additive relationship between age and education, with a reduced benefit of more education in older compared to younger participants. This might be a consequence of older participants having reduced cognitive and learning abilities that could not be overcome by educational experience (Blachstein & Vakil, 2016). The absence of interactions between education and other variables is further emphasised by the additive effects of education and other variables (e.g., initial severity and lesion size). The one exception to this is that the benefit of education was eroded in older participants with large left hemisphere lesions and severe initial symptoms. These participants likely sustained substantial damage to the brain regions that can support language after stroke making it much harder to improve or recover their abilities irrespective of education. Indeed, lesion size (whole brain and left hemisphere) was significantly larger in participants with Severe compared to Moderate and Mild initial severity (*p* = <0.001 for both) and in participants with Moderate compared to Mild initial severity (*p* = 0.001).

### C. The effect of education on recovery

The current study did not find any evidence that more education facilitated the degree of recovery from aphasia, using either long-term CAT scores, or self-reports of language change. This is consistent with a study of 34 participants by (de Riesthal & Wertz, 2004), but contrasts with the much larger study by Kim et al. (2019), which is the only one to demonstrate an effect of education on aphasia recovery. Future studies are now required to understand our null findings here. A key methodological issue is that our recovery measures were based on participants’, or their carers’ descriptions of their own abilities, typically guided by trained speech and language therapists. As ability was assessed retrospectively, the measures rely on both memory and insight which will undoubtedly result in measurement error, and could de-sensitise our study to the effect of education on recovery. Nonetheless, the same recovery measures demonstrated a significant benefit of clinical therapy on participant-reported recovery (Roberts et al., 2022) and we have unpublished work showing highly significant relationships between these ability measures and (i) lesion site and (ii) objective assessments from the Comprehensive Aphasia Test (CAT) taken at the same time point post-stroke.

A second major issue is that the CAT was typically conducted relatively late in the recovery phase (more than one year post-stroke). Given that many patients continue to recover beyond a year (Hope et al., 2017), a large proportion had reached ceiling level performance. This results in less variance than when assessments are conducted earlier (e.g., one year post-stroke as in Kim et al., 2019), making it more difficult to capture the impact of education on recovery. Ideally, we need to monitor the effect of education on recovery longitudinally, using exactly the same objective language assessments at regular time points throughout the first year and beyond.

Finally, other differences between Kim et al. (2019) and our study include the language spoken (Korean versus English), the language assessment (CAT versus the Korean version of the Frenchay Aphasia Screening Test) and cultural differences in education systems and attitudes to formal schooling. It would be interesting to observe if such factors influence the effect of education on language performance.

### Clinical and research implications for interpreting post-stroke language impairments

The sensitivity of the CAT to formal educational differences (according to this study’s definition) in stroke patients may have implications for clinical practice and for research. For clinical practice, there is a very small chance that assessment scores could be misinterpreted. However, this concern only applies to patients whose scores are within 2 to 4 points of the aphasia threshold, and is further diminished because clinicians’ decision-making and treatment planning already draws information from a range of assessments and observations, including patients’ functional abilities, self-reports, and impairments, as well as from social and demographic factors such as education – and so overall, the chance of misdiagnosis should be negligible. Nevertheless, we highlight the value of using participant-reported outcome measures to supplement objective assessment scores. For example, a patient with less education may self-report normal language when the CAT indicates mild aphasia. Conversely, a patient with more education may consider themselves to have aphasia due to subtle post-stroke language changes which go undetected on the CAT, or indeed other standardised language assessments.

The implications for research are likely to be more significant, because behavioural language data are more likely to be investigated in isolation of other participant characteristics. For example, task scores are often used to categorise participants either as having aphasia or not, or into different levels of aphasia severity. Plausibly, participants could be miscategorised because of their education amount, and erroneously treated as “recovered” when they in fact have mild impairments – or erroneously treated as mildly aphasic when their abilities were in fact back to normal. We found that up to 45% of participants fell within the score range that could be miscategorised as “aphasic” or “normal”. For example, for Naming, 122/749 participants scored from 60 to 65, which could be miscategorised if education amount is not considered. Ultimately, this leads to inaccuracies in analyses of outcomes and recovery.

Given these issues, this study advocates that, for both clinical practice and research, borderline CAT task scores should be interpreted in light of the amount of education a patient completed before their stroke; potentially using the “normative modelling” approach adopted by prior studies of neurotypical and stroke populations (Cavaco et al., 2013; Olabarrieta-Landa et al., 2015; Tombaugh et al., 1999; Zec et al., 2007). This approach provides a calculation to adjust any individual’s score for the influence of variables such as education, as well as age and sex. The resulting adjusted score indicates how that individual performed with respect to others with similar demographics to them (Casaletto et al., 2015; Shirk et al., 2011). As far as we are aware, no such approach has been implemented for the CAT. Considering how individual factors contribute to variation in CAT outcomes is in line with a personalised approach to assessment, management and prediction of outcome and recovery (Brady et al., 2022).

## Limitations and future directions

The measurement and definition of education has varied across prior studies, and there is currently no agreement as to what constitutes “more” or “less” education. In the current study, “years of formal education” was treated as a continuous variable, with “more education” defined as that typically undertaken after the age of 18; and “less education” defined as that typically done before the age of 18. The problem with both of these measurements is that they disregard the possible effect of different education settings or content, which may easily arise within, or across, countries, cultures, and generations (for example, academic versus vocational subjects). A more rigorous approach is therefore needed which incorporates more detail about education setting, content, and attainment, and considers other factors that may moderate education such as intelligence and learning difficulties, as well as traditionally non-academic learning experiences, such as job-specific training or professional development. Furthermore, this study did not measure socioeconomic status, whose close relationship with education compels further consideration of whether the apparent effect of education in fact reflects broader socioeconomic differences (Sheridan & McLaughlin, 2016). Indeed, the exclusion of apprenticeships and other “non-academic” learning from our definition of “formal education” may bias the sample. Geographically, we included more participants from London and the South East of England than from the North East of England where more education is less prevalent (Office for National Statistics, 2023). Future studies could investigate this by considering factors such as geographical area and occupational status.

A second and related limitation is that the current study was unable to control for all of the many factors that influence aphasia outcome and recovery, or that may interact with the effect of education. For example, the amount of speech and language therapy received may have influenced the speed of recovery, and/or interacted with the effect of education on recovery (Brady et al., 2016; Roberts et al., 2022). Real-world language practice, such as occupation, and other daily activities and interactions may influence recovery, if they draw on linguistic strategies (e.g., completing crosswords or reading for pleasure). Similarly, non-linguistic cognitive ability, measured in Johnson et al. (2022) using the WAIS Matrices subtest as an indicator of non-verbal problem solving and reasoning, has been correlated with education amount in prior studies. This may overlap with, or explain the effect of education, if for example non-linguistic skills are associated with more education.

A third limitation is the potential impact of excluding participants with missing CAT or participant-reported severity scores. If these data were missing because of severe impairments at one week, or pre-stroke illiteracy, then their exclusion could bias the sample towards those with milder initial impairments. However, it is more likely that the data were missing due to participants not being able to remember their early abilities. Conversely, missing CAT data are more likely to occur if participants were unable to complete a task due to aphasia severity. As only 3 participants had missing CAT data, the impact of their exclusion from the analyses is relatively small, and unlikely to inflate the effect of education.

A fourth limitation is that this study could not assess the impact of education on communication participation and quality of life. A previous study by Worrall et al. (2017) reported that more education was a negative determinant of participation, in 58 participants. It would be valuable to repeat the current study’s approach, using a variety of different aphasia outcome measures (including, for example, quality of life, participation and functional communication), as well as widening the range of social determinants of health that likely interact with the effect of education (O’Halloran et al., 2023).

Finally, by distinguishing participants who reported normal language in the first week post-stroke from those who reported aphasia, we were able to consider this group as “non-aphasic controls”, leading to the critical revelation that the CAT is sensitive to education amount, irrespective of the presence of absence of aphasia – as demonstrated by other studies of education effects in neurotypical people. This suggests that future studies should investigate the effect of other non-stroke factors (such as cultural/language background) on language performance.

## Summary and conclusions

Using a large sample of stroke survivors, we found that participants with more pre-stroke education had better performance on a range of language tasks from the Comprehensive Aphasia Test when assessed in the chronic stage of recovery. Moreover, the benefit of more education was found to be independent of (i.e., additive with) the influence of other variables known to impact language performance after stroke. Most strikingly, we did not find a greater benefit of education for aphasic compared to non-aphasic participants. To the contrary, education benefitted all participant groups – except older participants with severe aphasia and large strokes whose outcomes were not influenced by prior education. These results are inconsistent with education bestowing a protective effect on aphasia outcome. Likewise, there was no evidence that education plays a specific role in aphasia recovery. Instead, our results highlight how aphasia assessments are sensitive to the amount of pre-stroke education the participant has received.

The practical implication of the results is that aphasia clinicians and researchers should consider educational background when they interpret assessment scores. In addition, future studies should consider assessing whether education influences language abilities measured at earlier and more regular timepoints after a stroke, to determine whether a more subtle effect of education on recovery exists.

## Supplementary Material

Supplemental Material
