# Supplementary material for: The impact of pre-stroke formal education on language test performance in aphasic and non-aphasic stroke survivors
Source: Aphasiology. 2024 Dec 5;39(11):1480–502. doi: 10.1080/02687038.2024.2434864 (PMC12839798; doi:10.1080/02687038.2024.2434864)
Supplement: Supplemental Material [file PAPH_A_2434864_SM5002.docx]

**SUPPLEMENTAL MATERIAL**

**Table S1.** The benefit of more years of education on different language tasks in neurotypical adults.

| **Task** | **Study** | **Education definition** | **Language** |
| --- | --- | --- | --- |
| Naming | Neils et al. (1995), Roberts and Doucet (2011), Soylu and Cangöz (2018), Constantinidou et al. (2012), Rodríguez-Lorenzana et al. (2020) | ‘More’ education ranged from >9 to >13 years.  ‘Less’ education ranged from 0-4 to <9 years. | Turkish, French (Canada), English, Greek (Cyprus), Spanish (Ecuador) |
| Verbal fluency | Ratcliff et al. (1998), Brucki and Rocha (2004), Abdel Aziz et al. (2017), Rodríguez-Lorenzana et al. (2020), Van der Elst et al. (2006) | ‘Less’ education ranged from 0 to >12 years.  ‘More’ education ranged from 10 to 15 years. | Hindi, Portuguese (Brazil), Arabic (Egypt), Spanish (Ecuador), Dutch |
| Spoken picture description | Ardila and Rosselli (1996) | ‘Less’ education: 3-7 years.  ‘More’ education: >12 years. | Spanish (Colombia) |
| Digit span | Zimmermann et al. (2015), Choi et al. (2014) | Less education ranged from 0-3 to 5-8 years.  More education ranged from >9 to >13 years. | Portuguese (Brazil), Korean |

**Table S2.** The covariates factored out of each CAT analysis in addition to: sex, handedness, age at stroke, time between stroke onset and CAT (i.e. time post-stroke), left hemisphere lesion size, right hemisphere lesion size, and dyslexia status.

|  | Initial ability scores | | | Semantic memory | Vision status | Hearing status |
| --- | --- | --- | --- | --- | --- | --- |
|  | Speak  ing | Reading | Comprehension |  |  |  |
| **Overall language ability** | Yes | Yes | Yes | Yes | Yes | Yes |
| **Naming:** | Yes | - | - | Yes | Yes | - |
| Semantic fluency | Yes | - | - | Yes | - | - |
| Letter fluency | Yes | - | - | Yes | - | - |
| Object naming | Yes | - | - | Yes | Yes | - |
| Action naming | Yes | - | - | Yes | Yes | - |
| **Repetition:** | Yes | - | - | Yes | - | Yes |
| Word repetition | Yes | - | - | Yes | - | Yes |
| Complex word repetition | Yes | - | - | Yes | - | Yes |
| Nonword repetition | Yes | - | - | - | - | Yes |
| Digit span | Yes | - | - | - | - | Yes |
| Sentence repetition | Yes | - | - | Yes | - | Yes |
| **Reading:** | - | Yes | - | Yes | Yes | - |
| Reading words | - | Yes | - | Yes | Yes | - |
| Reading complex words | - | Yes | - | Yes | Yes | - |
| Reading function words | - | Yes | - | Yes | Yes | - |
| Reading nonwords | - | Yes | - | - | Yes | - |
| **Spoken picture description:** | Yes | - | - | Yes | Yes | - |
| Total number of words | Yes | - | - | Yes | Yes | - |
| Number of appropriate information-carrying words | Yes | - | - | Yes | Yes | - |
| Number of inappropriate information-carrying words | Yes | - | - | Yes | Yes | - |
| Grammatical well-formedness | Yes | - | - | Yes | Yes | - |
| Syntactic variety | Yes | - | - | Yes | Yes | - |
| Speed | Yes | - | - | Yes | Yes | - |
| **Spoken comprehension:** | - | - | Yes | Yes | Yes | Yes |
| Spoken word-to-picture | - | - | Yes | Yes | Yes | Yes |
| Spoken sentence-to-picture | - | - | Yes | Yes | Yes | Yes |
| Spoken paragraph | - | - | Yes | Yes | - | Yes |
| **Written comprehension:** | - | Yes | - | Yes | Yes | - |
| Written word-to-picture | - | Yes | - | Yes | Yes | - |
| Written sentence-to-picture | - | Yes | - | Yes | Yes | - |

**Table S3.** Amount of variance accounted for in each language outcome when education is the only regressor.

| **Language measure** | **R^2^** | **p value** |
| --- | --- | --- |
| **Naming** | 0.018 | <0.001* |
| Semantic fluency | 0.030 | <0.001* |
| Letter fluency | 0.041 | <0.001* |
| **Repetition** | 0.010 | 0.007 |
| Nonword repetition | 0.012 | 0.003 |
| Digit span | 0.011 | 0.005 |
| **Reading** | n/a | n/a |
| Reading nonwords | 0.012 | 0.003 |
| **Spoken picture description** | 0.012 | 0.003 |
| Total number of words | 0.018 | <0.001* |
| Number of appropriate information-carrying words | 0.015 | <0.001* |
| Number of inappropriate information-carrying words | 0.005 | 0.046 |
| Grammatical well-formedness | 0.016 | <0.001* |
| **Spoken comprehension** | 0.014 | 0.001* |
| Spoken sentence-to-picture matching | 0.014 | 0.001* |
| **Written comprehension** | 0.017 | <0.001* |
| Written sentence-to-picture matching | 0.016 | <0.001* |

**Legend:** Amount of variance accounted for by education (R^2^), and its significance (p value) for each summary score and task in linear regression analyses. Only the summary scores and tasks on which there was a significant effect of education when other variables were factored out (see Table 3) were included. n/a = not applicable because education was not significant on Reading in the main analysis. * = significant after correction for multiple comparisons (= 6 summary scores; 20 tasks).

**Table S4**. Interactions between Education and Age.

|  | 2-way interaction:  Ed & Age | Effect of education on: | | 4-way interaction:  Ed, Age,  LH & initial ability |
| --- | --- | --- | --- | --- |
|  |  | Younger | Older |  |
| Overall language ability | 0.049 | 0.004 | ns | ns |
| Naming | 0.038 | 0.001* | ns | 0.019 |
| Repetition | 0.050 | 0.042 | ns | ns |
| Reading | 0.020 | 0.020 | ns | ns |
| Spoken Picture Description | ns | 0.056 | ns | 0.030 |
| Spoken Comprehension | ns | 0.048 | ns | ns |
| Written Comprehension | ns | 0.013 | ns | ns |

**Legend:** p values for the two-way interaction between education and age (n=749), the main effect of education in younger (n=375) and older (n=374) participants, and the four-way interaction between education (Ed), age, left hemisphere lesion size (LH) and initial severity: for overall language ability and for each summary score. ns=not significant (p=>0.05 uncorrected). Due to missing data for some variables, 8 participants were excluded from the Naming analysis; 6 were excluded from the Repetition analysis; 1 was excluded from the fluency analyses and 177 were excluded from the Reading analysis (primarily because of missing data on initial reading ability). *=significant after correction for multiple comparisons.
